# GEOWEALTH-US: Spatial wealth inequality data for the United States, 1960–2020

**DOI:** 10.1038/s41597-024-03059-9

**Published:** 2024-02-28

**Authors:** Joel Suss, Tom Kemeny, Dylan S. Connor

**Affiliations:** 1 Bank of England, Threadneedle Street, London, EC2R 8AH UK; 2 International Inequalities Institute, London School of Economics, Houghton Street, London, WC2A 2AE UK; 3 Munk School of Global Affairs & Public Policy, University of Toronto, Toronto, M5S 3K7 Canada; 4 School of Geographical Sciences & Urban Planning, Arizona State University, Tempe, 85281 USA

**Keywords:** Geography, Sociology, Society, Economics

## Abstract

Wealth inequality has been sharply rising in the United States and across many other high-income countries. Due to a lack of data, we know little about how this trend has unfolded across locations within countries. Examining the subnational geography of wealth is crucial because, from one generation to the next, it shapes the distribution of opportunity, disadvantage, and power across individuals and communities. By employing machine-learning-based imputation to link national historical surveys conducted by the U.S. Federal Reserve to population survey microdata, the data presented in this article addresses this gap. The Geographic Wealth Inequality Database (“GEOWEALTH-US”) provides the first estimates of the level and distribution of wealth at various geographical scales within the United States from 1960 to 2020. The GEOWEALTH-US database enables new lines of investigation into the contribution of spatial wealth disparities to major societal challenges including wealth concentration, income inequality, social mobility, housing unaffordability, and political polarization.

## Background & Summary

Following a four decade period of sustained growth in wealth inequality in the United States, less than 10 percent of families now possess 70 percent of national wealth^1–3^. The trajectory of rising national wealth inequality resembles other unfavorable long-term patterns of income polarization and declining intergenerational mobility^4,5^. For historical income and intergenerational mobility dynamics, there is a growing realization that these prevailing trends have, in fact, arisen from a strongly differentiated subnational geography^6–11^. In contrast, we still know very little about the geography of wealth inequality and how it has changed over time.

This knowledge gap not only limits our understanding of broader societal trends in inequality, but also the social, economic, political, and even epidemiological consequences of concentrated wealth^12,13^. Specifically, wealth inequality has previously been linked to the local provision of public goods^14,15^, social mobility^7,16–18^, support for populism^19–21^, and the health of local economies^22,23^. Despite the role of wealth in giving rise to disparities in income^24^, wealth and income represent distinctive facets of economic inequality^25^, with potentially different roots and implications^26,27^. New evidence suggests that wealth is, in fact, a primary source of overall rising income inequality^28^. There is therefore a great need for focused investigation into the changing geography of wealth within and beyond the United States.

This article presents a new source of information on the long-term geography of wealth in the United States: The Spatial Wealth Inequality Database (“GEOWEALTH-US”)^29^. GEOWEALTH-US provides estimates of the level and distribution of wealth at various spatial scales within the United States from 1960 to 2020. The GEOWEALTH-US database not only enables new lines of investigation into the causes and consequences of wealth inequality in the United States, but also a flexible methodological framework for generating estimates of household wealth across space and time.

We overcome sizeable limitations in measurement and data availability to enhance our understanding of the changing geography of wealth. The stock of a households’ wealth is typically measured as the value of its assets net of total debts, across a range of asset types, such as cash holdings, real estate, and financial investments. Unlike income flows, which are reported in the census, few public data sources report on personal assets and debts, or on their constituent components. Our understanding of how individuals’ wealth in the U.S. has changed over time comes from ‘capitalization’ models of income information in confidential administrative data linked to taxes^24^, or more directly from a range of household surveys^26^. While each has advantages and disadvantages^1^, concerns around confidentiality and other issues mean that none of these data sources can be used to directly describe spatial disparities in wealth. The estimates provided in the GEOWEALTH-US database do not face confidentiality constraints and therefore enable the first granular spatiotemporal analyses of wealth for the contemporary United States.

The framework used to construct the GEOWEALTH-US database relies on the application of machine-learning-based imputation. Using rich survey information from the Federal Reserve’s Survey of Consumer Finances (SCF), we generate predictive models of household wealth using ensemble learning. We then use these models to impute wealth among households in Census population surveys that include geographical identifiers. The end result is a dataset that permits description of variation in wealth between places (‘geography of wealth’), as well as how wealth is distributed *within* urban and regional economies (‘local wealth inequality’). GEOWEALTH-US enables researchers to track the evolving geography of wealth and wealth inequality in the US between 1960 and 2020, across more than 700 local labor markets that span the entirety of the lower 48 states. The underlying imputation framework could be extended to generate estimates at finer spatial units (e.g., census tracts, incorporated places), for different countries, or to support efforts to estimate rates of intergenerational wealth transmission^30^.

Importantly, the estimates of wealth inequality provided in the GEOWEALTH-US database are derived from multidimensional measures of assets and debts. Our multidimensional approach marks a substantial advancement in the field since, at present, our understanding of the geography of wealth is predominantly limited to the housing market^31,32^. Home values and mortgage information are reported in several public data sources, including in tabulations and extracts of the decennial census. But, while important – especially for those who are less affluent – home values are only one among several channels through which wealth can vary across locations. Notably, both household debt and stock market participation rates in the United States are currently at all-time highs. In fact, home values and net wealth are now only moderately correlated across American households (*r* = 0.535, *p* < 0.001, based on authors’ calculations using Bureau of Labor Statistics’ Survey of Consumer Finances 2015–16 data). The GEOWEALTH-US database therefore provides insight on wealth inequality, as measured across a broad, and increasingly diversified, range of assets and liabilities^33^. More broadly, this work contributes to the ongoing efforts to utilize big data and computation to describe, understand, and ultimately address patterns of inequality and inequity^34,35^.

**Fig. 1:**
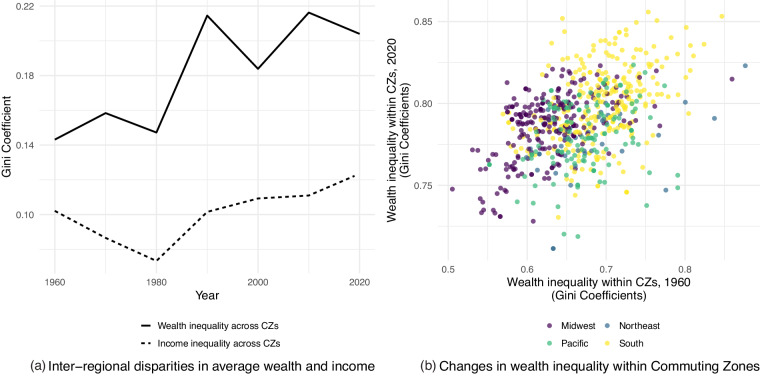
The geography of wealth and local wealth inequality, 1960–2020. For the period 1960–2020, panel (**a**) uses Gini coefficients to describe trends in inter-regional inequality in terms of average household income and wealth across U.S. commuting zones, defined using 1990-vintage commuting flow data. Wealth estimates come from the GEOWEALTH-US dataset that is the primary output of this study. Income series is from Kemeny and Storper^8^. Note that this panel shows that wealth gaps between places has grown much more sharply than income gaps. Panel (**b**) visualizes the correlation between 1960 and 2020 measures of local wealth inequality – that is, levels of household wealth inequality within each commuting zone, again measured using Gini coefficients. The positive but only moderately strong correlation between local wealth inequality suggests a mix of continuity and turbulence in the ranks of more and less wealth-unequal locations in the United States.

Our initial investigation of the spatial and temporal patterns of the GEOWEALTH-US database reveals three key features of the changing geography of wealth in the United States. First, Fig. 1a reveals that the distribution of wealth *between* regions has become meaningfully more unequal since 1970. US wealth holdings have become increasingly concentrated in a smaller set of regions. Second, inter-regional *wealth* disparities have grown much larger than inter-regional *income* disparities, with income gaps growing trough-to-peak by 42 percent and wealth by 97 percent. This supports the intuition that spatial wealth inequalities require investigation over and above the study of income inequality. The sharp exacerbation of wealth inequality over this period make this a particularly urgent topic for further research. Third, Fig. 1b reveals patterns of both turbulence and persistence in the distribution of wealth *within* regions since 1960. Economies in the South are particularly notable in having high levels of wealth inequality in both 1960 and 2020. In the Midwest, however, wealth in 1960 was relatively evenly distributed within locations but has become much more unequal over the subsequent six decades. The worsening of inequality in the Midwest over this period is consistent with findings from studies of regional income inequality^8,36^ and intergenerational mobility^7^, suggesting interdependence and common underlying sources affecting different facets of spatial inequality. Our publication of the GEOWEALTH-US database provides new avenues for investigating the causes, consequences, and common coherence of these patterns.

## Methods

We start from the public-release files of the Federal Reserve’s Survey of Consumer Finances (SCF) that span the 1989–2019 period. Making use of the multiple household demographic and income attributes present in SCF, we predict household total wealth, gross assets and debts for households in successive waves of public-use Decennial and American Community Survey (ACS) microdata from the U.S. Census Bureau, obtained from IPUMS^37^. Using Census-derived information on household location, we generate estimates of the sub-national geography of residential net wealth and wealth inequality for various spatial units, including metropolitan areas and commuting zones.

In the absence of directly-observed, geographically-identified wealth data, our approach provides new opportunities to draw inferences about the spatial distribution of wealth. Like other surveys that record information on wealth, such as the Survey of Income and Program Participation (SIPP), SCF includes relevant demographic correlates. Unlike SIPP and other surveys, however, SCF also includes detailed information on household wealth and incomes. Crucially, SCF-based variables capture distinct categories of income, including wages, investment, and business income, closely matching the information contained in the Decennial Census and the American Community Survey. By observing relationships between income and wealth levels in the SCF, our imputation procedure is not exclusively bound to sociodemographic characteristics, which we know to be limited in their predictive power of income and wealth differences across locations. Put another way, the inclusion of income data enriches our understanding of wealth differences among individuals with similar demographic and educational profiles in different locations^38^. Thus, by capturing not just demographics, but also detailed income information, housing tenure and value, our model is best situated to generate accurate predictions of spatial disparities in wealth.

Beyond the SCF’s unparalleled direct detail about joint components of wealth and income, these data offer a useful basis for prediction because of their coverage of households across the full range of the wealth distribution^26^. Many national household surveys have a missing top wealth problem^39^, prompting alternative approaches that ‘capitalize’ income information in administrative tax data^2,40,41^. But in the modern (post-1983) SCF, major oversampling for rich households has been demonstrated to effectively cover the top of the wealth distribution^42,43^. This represents a key difference between the SCF and otherwise comparable surveys for parts of Europe and the UK^43^. Meanwhile, the SCF describes wealth for households whose housing-centered assets are not sources of taxable income, as well as low-wealth households that may pay little or no taxes^1^.

Like the income capitalization method, our approach to estimating wealth inequality relies on first imputing household wealth. However, relative to tax data, coarser categories of income available in the ACS/Census make imputation using the capitalization method inefficient. A strength of our approach is that we build a sophisticated statistical model of household wealth using all available variables in common between the SCF and Census; we therefore sidestep the problem of assuming a fixed rate of return^44^, which might reasonably vary by household characteristics and income level. Given the finer spatial scales at which we are interested in estimating wealth inequality, minimizing the prediction error at the household-level is important, whereas, as Saez and Zucman^2^ note, the income capitalization method is noisy at the individual-level, but this matters less at when pooling many thousands of families at the country-level. A comparison of the efficacy of our approach with the income capitalization method would be enriching, and we encourage this as a point for further research with appropriate data.

Our construction of the data comprises four steps: (1) build a model of household wealth using the SCF; (2) predict wealth using Census population survey data; (3) address top wealth holders in the Decennial Census and American Community Survey using Pareto tail estimation; and (4) estimate wealth and wealth inequality for widely used spatial units (e.g., commuting zones).

### Step 1: Build a model to predict household wealth

Using the SCF data, we build and combine a set of stacked ensemble models (‘ensemble combination’) to arrive at the most accurate available predictions of household wealth. As a general approach, stacking involves combining a number of predictive models^45,46^. Typically, a set of base (or Level 1) models are trained on a subset of the data. A second-level model is then fit on a separate subset of the data, using the Level 1 predictions as inputs. The aim is to garner improvements in prediction that result from bringing together a diverse (relatively uncorrelated) and accurate set of models.

We chose to use stacked ensembles based on a careful comparison between different potential approaches. We evaluated the ensemble combination relative to alternative treatments of net wealth, including modelling the inverse hyperbolic sine transformation of net wealth, and taking the net difference between models separately predicting gross wealth and debts. We also evaluated the performance of each ensemble relative to the individual constituent models that comprise it. Details of comparisons between our preferred approach and other models are found in the first part of our Technical Validation section; these demonstrate that our stacked ensembles jointly outperform available alternatives.

Our stacked ensemble is made up of seven base models: generalized linear regression (GLM), elastic net regression (EN), random forest (RF), extreme gradient boosted trees (XGB), neural network (NN), support vector machine (SVM), and K-nearest neighbors (KNN). Note that some of these ‘standalone’ models are themselves ensembles. In particular, random forests are known as ‘bagged ensembles’ – bootstrapped aggregations of individual decision or regression trees, while boosted models are ensembles of sequentially grown trees^47,48^. For the Level 2 model, we estimate a simple linear regression.

To produce a final predicted value of wealth for each household, $\widehat{Net\,Wealth}$, we combine the outputs of four separate ensembles. First, we estimate the probability of having positive wealth (*P*(*Wealth* > 0)); 8.9% of households in the SCF sample have either no wealth or are in debt. If the predicted probability is at or above the decision threshold (which we select based on the maximum Kappa value when varying the threshold from 0 to 1 by 0.025 on the test sample), the fitted value of a positive wealth ensemble model (${\widehat{Y}}_{+}$) is chosen, built using only data on households with some positive value of wealth. If the predicted probability is below the threshold, a further binary stacked ensemble predicts whether the household in question has zero or some quantum of negative wealth (*P*(*Wealth* > 0)). If the latter, a stacked ensemble estimates the quantum (${\widehat{Y}}_{-}$), modeled using data restricted to households with negative wealth. Formally:

1 $$\widehat{Net\,Wealth}=\left\{\begin{array}{lll}{\widehat{Y}}_{+} & {\rm{for}} & P(wealth > 0)\\ 0 & {\rm{for}} & P(Wealth\le 0)\;{\rm{and}}\;P(Wealth=0)\\ {\widehat{Y}}_{-} & {\rm{for}} & P(Wealth\le 0)\;{\rm{and}}\;P(Wealth\ne 0)\end{array}\right.$$

**Fig. 2:**
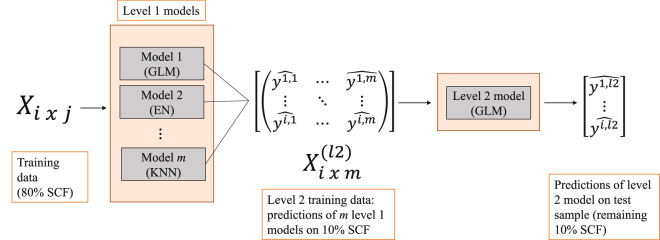
Structure of stack ensemble. The ensemble ‘stacks’ a level 2 (regression) model on top of the predictions of the level 1 models. All level 1 models are trained on a random 80% subset of the SCF (matrix *X*, with rows *i* and columns *j*, where *j* is the number of predictors available). Then level 1 predictions ($\widehat{{y}^{i,m}}$) are made on a separate 10% of the SCF, which becomes training data for the level 2 model (matrix *X*^*l*2^ with rows *i* and columns *m*, where *m* is the number of level 1 models). Finally, performance is evaluated for all level 1 and level 2 models on the remaining 10% of the SCF (the ‘test’ sample).

To fit the ensembles, we train Level 1 models on a random 80% of the SCF data (N = 42,748), and the Level 2 regression model on a validation set consisting of 10% (N = 5,341). We then evaluate performance on a test set consisting of the remaining 10% of data (N = 5,341). For Level 1 models which have hyperparameters to select (i.e. EN, RF, XGB, NN, SVM and KNN), in each fold we employ 5-fold cross-validation with random grid search (length of 10). For binary classification models - those modelling whether a household has positive wealth (*P*(*Wealth* > 0)) or no wealth (*P*(*Wealth* > 0)) - we up-sample the minority class within each cross-validation fold such that there are a balanced number of cases. That is, we sample with replacement from the subset of observations with negative or no wealth to ensure a 50/50 split. This up-sampling accounts for the imbalance in the outcome classes (e.g., more than 90% have greater than zero wealth). Figure 2 depicts the stacked ensemble structure.

#### Variable selection and transformations

To build the ensembles, we select the set of variables that are available in both the SCF and the Decennial Census and ACS. The complete set of variables (the ‘full model’) is available from 2008 onwards in the ACS and from 1989 onwards in SCF. Table 1 describes these inputs, listing variable names from the ACS, as well as corresponding variables in the 2019 SCF. Since our main estimated results for Census years 1960–1980 are based on the model of household wealth using data from 1989 onwards in the SCF, as a sensitivity check we also build models based on SCF+^1^, which harmonizes public-use SCF data with 1949–1983 SCF surveys. Models using SCF + perform worse on test samples, likely a result of the smaller, less-detailed set of variables available consistently from 1949 onwards. We also test whether models fit on data post-1983 data perform worse on a pre-1989 test sample, perhaps because relationships between our predictors and net wealth change over time. We find that the performance results are qualitatively similar on this out-of-time sample.

**Table 1:** Census and SCF variables and definitions by category.

Category	Variable (Decennial/ACS)	Variable (SCF)	Description
*Housing information*			
	VALUEH	houses	Value of primary residence
	OWNERSHP	houses, hdebt	Housing tenure – own outright, own with a mortgage, or rent
	MORTAMT1	paymort1	Monthly payment for 1st residential mortgage
	MORTAMT2	paymort2	Monthly payment for 2nd residential mortgage
	TAXINCL	x810	Whether tax is included as part of mortgage payment
	INSINCL	x810	Whether insurance is included as part of mortgage payment
	PROPTX99	x721	Annual property tax amount
	RENT	rent	Usual monthly rent
*Detailed income information*			
	INCWAGE	x5702	Wage and salary income
	INCBUS	x5704	Business income
	INCSS	x5722	Social security income
	INCWELFR	x5716, x5720, x5724, x5725	Income from welfare receipts
	INCINVST	x5706, x5708, x5710, x5714	Investment, interest and dividend income
	INCRETIR	x5724, x5725	Retirement income, e.g. IRA and 401k
	INCOTHER	x5712, x5718	Other income not included in available categories
*Demographic information*			
	AGE	age	Age
	RACE	x6809	Race
	EDUC	x5901, x5902, x5904, x5905	Educational attainment
	SEX	hhsex	Sex
	MARST	x8023	Marital Status
	FAMSIZE	x101	Number of own family members in household
	YEAR		Year
*Employment information*			
	OCC	x7401, x7411	Occupation
	IND	x7402, x7412	Industry
	EMPSTAT	x4100, x4700	Employment status
	CLASSWKR	x4106, x4706	Class of worker
	UHRSWORK	x4110, x4710	Usual hours worked per week
	WKSWORK2	x4111, x4711	Weeks worked last year, intervalled
*Other information*			
	VEHICLES	nvehic	Number of vehicles available
	HCOVANY	x6341	Any health insurance coverage

Note: Detailed definitions of variables available at https://usa.ipums.org/usa-action/variables/group. Codebook for SCF (2019) found at https://www.federalreserve.gov/econres/files/codebk2019.txt. Samples in the SCF vary from a low of 3,143 in 1989 and grow to a high of over 6,000 from 2010. These figures do not account for the implicates provided for each sampled household. For fuller details on the construction of the SCF, consult^71,72^.

Certain potentially relevant variables were not measured in earlier years of the Decennial Census. For years in which the full ensemble cannot be estimated due to variable unavailability, we build unique ensembles using the subset of variables that are available in those years. Table 2 provides a breakdown of variable availability over the study period.

**Table 2:** Census variables and availability by year.

Variable (Decennial/ACS)	2020	2010	2000	1990	1980	1970	1960
*Housing information*							
VALUEH	X	X	X	X	X	X	X
OWNERSHP	X	X	X	X	X	X	X
MORTAMT1	X	X	X	X			
MORTAMT2	X	X	X	X			
TAXINCL	X	X	X	X	X		
INSINCL	X	X	X	X	X		
PROPTX99	X	X	X	X			
RENT	X	X	X	X	X	X	X
*Detailed income information*							
INCWAGE	X	X	X	X	X	X	X
INCBUS	X	X	X	X	X	X	X
INCSS	X	X	X	X	X	X	
INCWELFR	X	X	X	X	X	X	
INCINVST	X	X	X	X	X		
INCRETIR	X	X	X	X			
INCOTHER	X	X	X	X	X	X	X
*Demographic information*							
AGE	X	X	X	X	X	X	X
RACE	X	X	X	X	X	X	X
EDUC	X	X	X	X	X	X	X
SEX	X	X	X	X	X	X	X
MARST	X	X	X	X	X	X	X
*Employment information*							
OCC	X	X	X	X	X	X	X
IND	X	X	X	X	X	X	X
EMPSTAT	X	X	X	X	X	X	X
CLASSWKR	X	X	X	X	X	X	X
UHRSWORK	X	X	X	X	X		
WKSWORK2	X	X	X	X	X	X	X
*Other information*							
VEHICLE	X	X	X	X			
HCOVANY	X	X					

Note: Detailed definitions of variables in the Decennial and American Community Survey available at https://usa.ipums.org/usa-action/variables/groupusa.ipums.org/usa-action/variables/group.

Before splitting the data between training, validation and test samples, we transform and harmonize a subset of the variables in the SCF dataset. In particular, we use the inverse hyperbolic sine transformation for continuous variables which have a pronounced right-skew and contain zeroes. This issue mainly applies to the home value and income variables. We also harmonize several of our categorical variables by aggregating values into a coarser set of common categories (e.g. education, occupation, and industry). We do not remove any observations that might in other circumstances be considered outliers; such extreme values are potentially important in our context, particularly for the identification of very wealthy or high-earning households.

#### Variable importance

Although machine learning approaches to prediction tend to outperform frameworks based on simple linear regression, the opacity of the underlying decision process can pose problems. To address this issue of ‘weak explainability’, we investigate whether the most influential variables within the models have intuitive meaning in terms of predicting wealth. To quantify the importance of each variable, we compute Shapley values for each test set observation, a well-known approach to model interpretability that originated from research in game theory^49^. Shapley values provide a local interpretation gauging the relative importance of each variable for each specific prediction. We can then take the mean absolute Shapley value per variable to make global assessments of importance.

**Fig. 3:**
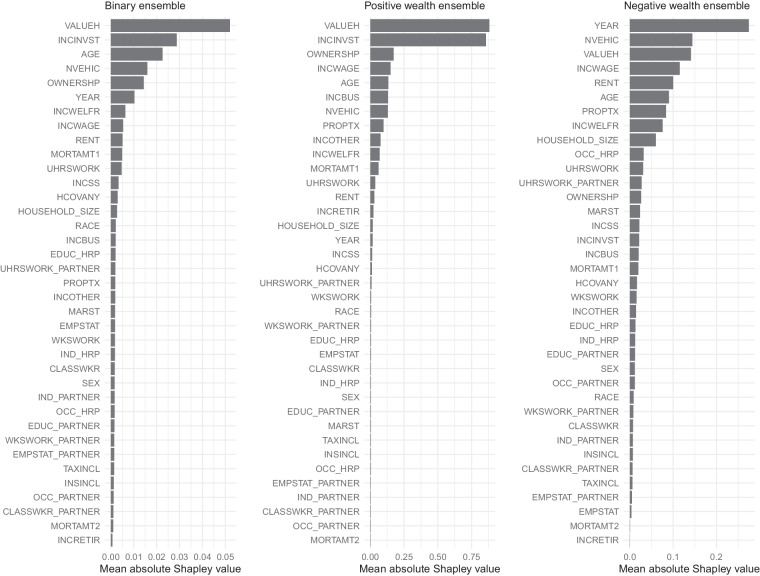
Mean absolute Shapley values for ensemble combination. Displayed are mean absolute Shapley values for household variables with predictive power on household wealth, with higher Shapley values indicating greater predictive power. Here we include Shapley values for separate predictions of the binary ensemble (whether positive or negative wealth); and individual models predicting levels of positive or negative wealth.

Figure 3 presents these results separately for the dominant model (random forest) for the main components of the ensemble combination – the binary, positive wealth, and negative wealth ensembles. The figure rank orders the importance of each input variable across our three models. Home values (VALUEH) are the most important variable for the binary and positive wealth ensembles followed by investment income (INCINVST). However, the variables of importance to the negative wealth ensemble look quite different. Specifically, the year of observation in the Census is the single most important predictor, followed by vehicle availability (NVEHIC) and again home values (VALUEH). The relationship between year of observation and negative wealth is a reflection of the rising levels of personal debt over recent decades.

**Fig. 4:**
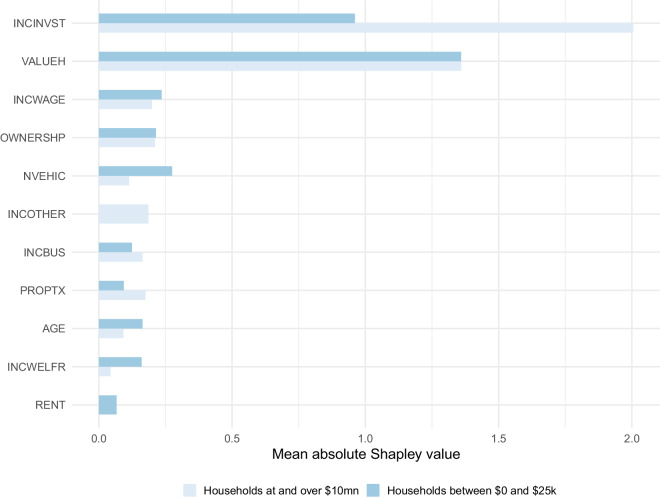
Mean absolute Shapley values for high- and low-income households. Displayed are mean absolute Shapley values for household variables generated from the positive wealth ensemble on sub-samples of the test data. Higher values indicate greater variable importance. The figure compares variable importance for households with income between 0 and $25,000, and at and over $10 million.

Because Shapley values give us local (i.e. prediction-by-prediction) information on explanatory power, we can also investigate how characteristics may contribute differently to wealth and debt depending on a household’s net worth. This is especially pertinent in our context, as we anticipate substantial variability in the nature of asset and debt accumulation across the distribution of wealth. In Fig. 4, we report mean absolute Shapley values for high and low net-worth households separately, defined as those with $10 million or more and between $0 and $25k respectively. When comparing these two groups, we observe that investment income is of greater consequence among high net worth households, whereas home values matter more at lower wealth levels. This fits with evidence from Saez and Zucman^2^ that finds that increases in investment income are primarily responsible for increases in top wealth shares. We also see that rent is an important predictor for only the low net-worth households, whereas income derived from other sources is an important predictor for high net-worth households.

### Step 2: Predict wealth using Census population survey data

Armed with trained stack ensembles, for the years 1960-2020, we then impute wealth for each household observed in the Census microdata. As noted above, we train year-specific ensembles to impute wealth in order to account for inconsistencies in the availability of variables across census years. We first limit the Decennial/ACS data to exclude those who are institutionalized or in group quarters. To match SCF, we measure demographic information using the household head. We then adjust all income and housing values to account for inflation, matching the SCF by bringing these to 2019 dollars.

Prior to imputation, we address top-coding issues in the Census microdata. These data include many variables that are top-coded to preserve respondents’ anonymity. For our purposes, relevant top-coded variables are: MORTAMT1, MORTAMT2, PROPTX, VALUEH, RENT, INCWAGE, INCBUS, INCSS, INCWELFR, INCINVST, INCRETIR, INCOTHER. Given the evidence on bias in measuring inequality resulting from such censoring^50,51^, for each of these variables we undertake procedures to adjust top-coded values. In broad terms, we take the following steps: i) use the SCF to derive a new maximum value and to estimate Pareto tail parameters, and ii) to top-coded census observations, we assign values from the estimated Pareto distribution based on the results of a ranking model built using SCF data. More specifically, as the SCF is not top-coded and over-samples rich households, we use the maximum value contained therein as an estimate of the maximum value in the population. For each decade and top-coded variable, we take the maximum from the combined SCF surveys covering the year in question and the preceding five years. For years prior to 1989, we use the maximum values from the SCF+^1^. We also use the SCF to estimate Pareto parameters for the distribution of values above the top-code. Using Kolmogorov-Smirnov (KS) statistics, we first test whether the distribution above the top-coded value is best approximated as Pareto or some other distribution, either log-normal or generalised Beta of the second kind. In all but a few cases, where the sample size used to estimate the Pareto tail parameter is small, Pareto distributions are the best fit.

From 1990 onward, the Decennial/ACS provides State-specific top-code thresholds for many variables, as well as the median or mean value above the listed threshold (or 99.5th percentile, depending on year). Given this additional information, we allow the Pareto tail parameter and maximum value to vary by State in order to minimize the distance between the mean of the estimated Pareto distribution and the State mean or median.

We then sample from the estimated Pareto distributions to adjust the values that are top-coded in the Census data, assigning these conditional on other household characteristics. We build a model using SCF data to rank top-coded observations in order of highest value. For this task we use the LambdaMART pairwise ranking algorithm, implemented with extreme gradient boosted trees^52^, which has been shown to perform relatively well in other broadly analogous situations, such as information retrieval for internet search^53^. As we may have multiple predictors censored simultaneously, the ranking model is more reliable for our purposes than predicted magnitudes exceeding the top-code quantum. In order to account for variability in rank, we take the average normalized predicted rank for 10 fits of the ranking algorithm (i.e. 10 configurations of hyperparameters chosen at random from a large grid). The highest ranked observation in the Census data is assigned the highest value from the estimated Pareto distribution, and so on. The assignment of above-top-code values is thus conditioned on the values of the other predictors in the model.

To validate this approach, we regress our imputed rank in the Decennial/ACS on the most granular geographical identifiers available (PUMA and county groups). The Adjusted-*R*-squared of these regressions are substantial, averaging 0.235 for 2020. This implies that there is strong spatial variability in our adjustment of values above the top-code, even though the absence of geographic identifiers in the SCF prevents us from incorporating them into the fitting of the ranking model.

After adjusting for top-coding, we impute wealth levels for the households in the Census data. To gauge the function of these adjustments, for the year 2020, Fig. 5 reports State-specific ACS-based Gini coefficients, comparing those in which top-codes are not adjusted, to those obtained by utilizing the Pareto distribution and a ranking algorithm for estimating and assigning values at and above the top-codes. As expected, this figure demonstrates importance of the top-code adjustment for the predictions of wealth levels and our estimates of inequality.

**Fig. 5:**
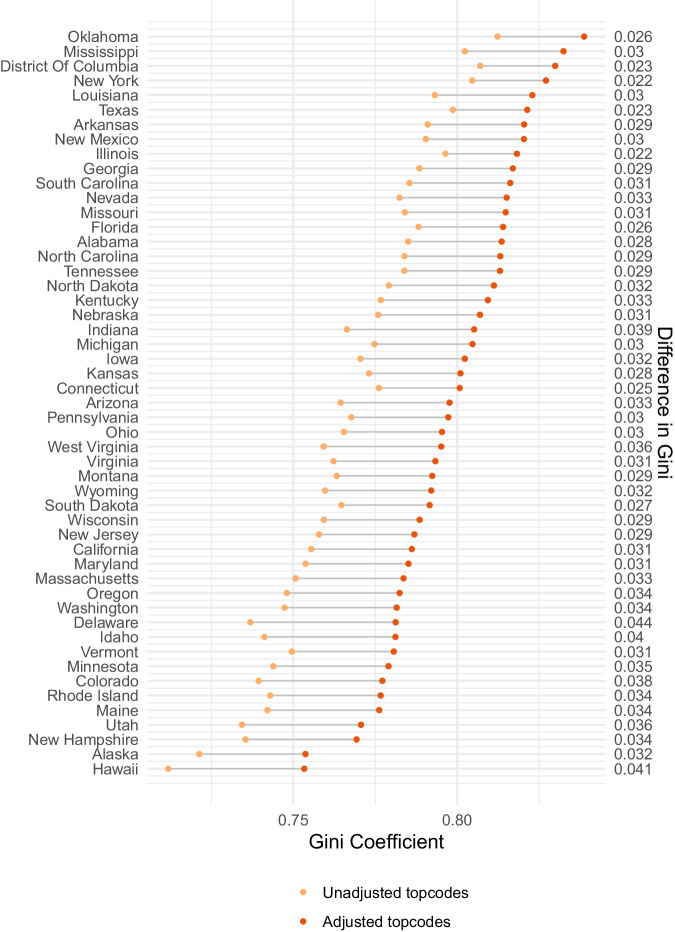
Wealth inequality estimates before and after topcode and bottomcode adjustments, 2020. Displayed is the impact on the estimated State-specific Gini Coefficients measuring household wealth when adjusting top and bottom-coded household variables in the Census/ACS. The right-hand axis for the figure provides the difference in the Gini Coefficient between adjusted and unadjusted estimates.

### Step 3: Address top wealth holders in the decennial census and american community survey

Given their significance to wealth inequality and their rarity in the population, extremely wealthy households require focused attention. The main concern is under-coverage at the top of the wealth distribution, which has multiple sources^54^. These include differential unit nonresponse, where wealthy households may be less likely to participate in surveys regarding their economic circumstances, and systematic underreporting, whereby wealthy households may be more likely to underreport the value of their assets^39^.

In response to such concerns, prior to estimating the geography of wealth and wealth inequality, we replace the top tail of the observed wealth distribution with a Pareto distribution^43,54–58^. We use the imputed Decennial/ACS wealth data as a basis to estimate year-specific Pareto tail parameters, *α*, using the maximum likelihood estimator^58^. Because estimates of *α* depend on the minimum threshold above which the distribution is considered to follow Pareto, we take an average of the estimated tail parameters across different thresholds^43^. In particular, we set thresholds at $500k, $600k, $700k, $800k, $900k, $1mn, $2mn, $3mn, and $5mn. Due to rising numbers of wealthy households over time, we use an additional threshold of $10mn for the 2010 and 2020 years of observation. We then replace the existing top tail with the estimated Pareto distribution.

Other sources of data on extreme wealth can also be incorporated into our approach. Earlier studies on wealth have sought to mitigate differential nonresponse bias by pooling survey data with household information drawn from ‘rich lists’ such as the Forbes 400, which comprises the top 0.00025 percent of wealthy households^43,59^. These households are excluded from the SCF due to identifiability concerns, and so may limit the maximum amount that can be imputed based on models of SCF household wealth, although in practice the ensemble models can extrapolate beyond the range of the observed data. For robustness, we added households from the Forbes 400 list to our SCF-derived imputed Census data. When we do so, we find that the impact on our estimates of *α* are negligible. This null result is consistent with earlier findings that estimates based on the application of Pareto tail procedures to the post-1983 waves of the SCF, where wealthy households are oversampled, yields very similar results to those that include Forbes 400 households^43^. We conclude that the exclusion of these households from the SCF is not a meaningful source of bias in our work.

### Step 4: Estimate wealth and wealth inequality at various spatial scales

As a final step, we use the imputed Census wealth data to estimate inequality levels at varying levels of geography. When doing so, we use Census-provided household weights.

We generate measures of local wealth inequality, including Gini coefficients and wealth shares, as well as mean and median wealth per area, at multiple spatial scales: PUMAs, 1990 Commuting Zones (CZs), Metropolitan Areas, States, Regions, and the country as a whole. PUMAs or broad equivalents (County Groups, State Economic Areas etc.) are available for the 1960 census and from 1990 onwards. We use the crosswalks provided by Dorn^60^ to infer a households’ CZ of residence based on their reported PUMA or equivalent. This requires multiplying the household weights by a factor which represents the probability that a household belongs to a given CZ (which is 1 where PUMAs or county groups lie entirely within a CZ, and less than 1 when split across multiple CZs). Table 3 provides details of data availability at different spatial scales.

**Table 3:** Datasets and coverage.

Dataset Name	Spatial Units	Unique Units	Observations	Years
puma_wealth_inequality.csv	Public Use Microdata Areas	11805	8592	1960–2020
cz_wealth_inequality.csv	Commuting Zones	741	5174	1960–2020
metarea_wealth_inequality.csv	Metropolitan areas	573	1677	1960–2020
state_wealth_inequality.csv	States	51	350	1960–2020
division_wealth_inequality.csv	Divisions	9	63	1960–2020

Given the imputation procedures used to estimate local wealth levels and distributions, it is appealing to capture the uncertainty around these estimates. To do so, we bootstrap a distribution of 100 inequality estimates, sampling with replacement from the distribution of imputed household wealth, using a 5% confidence level. A main advantage to this simulated approach is that it does not require any assumptions regarding the normality of the distribution of inequality estimates^61^.

Comparisons of the resulting dataset against published information on wealth for the United States are reported in the Technical Validation section.

## Data Records

Subnational-scale GEOWEALTH-US data^29^ are available on an open access basis through a Creative Commons Attribution 4.0 International (CC BY 4.0) license (https://creativecommons.org/licenses/by/4.0/creativecommons.org/licenses/by/4.0/). The data are hosted by the Inter-university Consortium for Political and Social Research (ICPSR) at 10.3886/E192306.

The data are organized into a series of individual comma-separated files (csv), with each file corresponding to a particular spatial unit of observation: state; 1990-vintage commuting zone; metropolitan area; PUMA; and division. Coverage over time is dependent on spatial units, per Table 3. The data at ICPSR include a brief metadata file in pdf format.

The primary variables in each of the GEOWEALTH-US datasets include locational identifiers (unique identifying codes and names of places); the number of Census households surveyed in that location; measures of central tendency (means and medians) for wealth; measures of spread for wealth (standard deviation); ratios of wealth at specific percentiles (for instance the ratio of wealth at the 90th/50th percentiles); as well as selected key demographic features of locations derived from the Census. For wealth estimates, we also include upper and lower bounds, based on the bootstrapping procedure described in Step 4 of the text.

## Technical Validation

In this section, we present exercises undertaken to validate the technical quality of the dataset. Three kinds of validation are reported. First, we describe the performance analysis on the SCF test sample used to arrive at our final model of wealth. Second, we conduct out-of-sample validation to verify that our model performs well in predicting household wealth using data beyond the SCF. Third, we report comparisons between our national and state-level inequality estimates and those obtained from other sources.

### Performance evaluation, SCF test sample

In order to select our final model of household wealth, we run a ‘horse race’ to evaluate competing approaches in terms of predictive performance. The winner is the approach which dominates in providing the most accurate predictions, as assessed by the true values observed in our SCF hold-out sample – the 10% test sample not used for fitting any model.

As reported in Step 1 of the Methods section, we fit a variety of well-known models and architectures, also exploring combinations of these in a stacked ensemble. We also consider the implications of different transformations of the outcome measure. Specifically, we explore outcomes as follows:

- ‘ENS’: binary model predicting whether a household has positive wealth; a model that predicts positive wealth; a model that predicts negative wealth; and a binary model whether a household has zero or some negative value of net wealth – see Eq. 1 above.
- ‘IHS’: the inverse hyperbolic sine transformation of net wealth
- ‘WD’: the net difference of separate models predicting gross wealth and debt

We test these alternative approaches in order to arrive at a final predicted net wealth value that adequately captures the proportion of households with zero or negative net wealth, while also accurately predicting quantitative values of net wealth.

To compare these permutations, we consider a number of performance metrics. First, we evaluate the ability of each approach to discriminate between households with positive, negative and zero net wealth. We do this through binary comparisons of households that have positive values of net wealth versus those that do not. In terms of the discriminant ability of our models, we focus on three key measures: the Brier score, the Kappa statistic, and the Area Under the Receiver Operating Characteristic Curve (AUC). For illustrative purposes, we report three further statistics: (1) overall accuracy, which describes the proportion of correct cases; (2) the true positive rate (TPR) that captures the proportion of households that are correctly predicted to have positive net wealth; and (3) the true negative rate (TNR), which describes the proportion of households that are correctly predicted to have negative net wealth. While the accuracy measure is typically used to measure performance, in situations where there is a large imbalance between classes – such as our own – it can be misleading. Specifically, the result can be high levels of accuracy while the minority class is not well predicted. The TPR and TNR help reveal whether there is an imbalance in accuracy across different outcome classes. The Kappa statistic overcomes the insensitivity to imbalance, comparing the observed accuracy versus the expected accuracy that would result from random change. AUC provides the probability of correctly discriminating between classes for a randomly selected observation, and is therefore also sensitive to imbalance. The Brier score – which is simply the difference between predicted probability minus the actual outcome (1 or 0) squared – is threshold-agnostic, and therefore provides an indication of the quality of a model’s predictions. For continuous predictions, we focus on the root mean squared error statistic (RMSE).

Table 4 reports these indicators for the full model. Broadly, we observe the strongest performance for the stacked ensemble (ENS) approach. The ENS has the lowest Brier score and the highest Kappa (at the optimal decision threshold). The benefits of the ensemble are evident when looking at the performance of the RF and XGB models, which are the top performing level one models on the Brier score and Kappa statistic, respectively. The binary ensemble encompasses the benefits of these two approaches while also minimizing their respective deficiencies (i.e. the poor Brier score for XGB, and relatively low Kappa for RF). We can also observe why the ensemble combination outperforms the single ensemble, where net wealth is transformed using the inverse hyperbolic sine (IHS) or when gross wealth and debts are modeled separately (WD). In these cases, overall accuracy is high but the approaches are insensitive to zero or negative wealth values, with each overlooking true negative cases (i.e. TNR = 0%). Note that an ensemble which models the raw, untransformed value of net wealth performs similarly to inverse hyperbolic sine approach. Equally, we find a similar pattern of performance when we do not transform either the outcome or any of the continuous predictors. Similar patterns are evident for the positive wealth models – ENS has the lowest RMSE for positive wealth. The negative wealth ensemble model underperforms by comparison to several of the level one learners.

**Table 4:** Comparison of performance across models for the full model (i.e. 2010–2020 variables).

Model	Brier	Kappa	AUC	Accuracy	TPR	TNR	RMSE ( + )	RMSE (−)
ENS	0.058	0.426	0.901–0.921	0.887	0.910	0.637	0.999	1.480
IHS	0.282	0.000	0.639–0.686	0.916	1.000	0.000	12.953	15.200
WD	0.078	0.000	0.538–0.566	0.916	1.000	0.000	1.351	19.239
GLM	0.146	0.394	0.884–0.907	0.877	0.900	0.623	1.273	1.475
EN	0.146	0.392	0.883–0.906	0.901	0.939	0.480	1.320	1.457
RF	0.064	0.396	0.887–0.909	0.879	0.903	0.617	1.030	1.457
XGB	0.129	0.420	0.901–0.921	0.887	0.910	0.626	1.003	1.458
NET	0.148	0.384	0.882–0.905	0.863	0.880	0.677	1.064	1.744
SVM	0.145	0.361	0.864–0.89	0.875	0.904	0.558	1.309	1.490
KNN	0.209	0.316	0.84–0.871	0.814	0.821	0.740	1.894	1.505

Note: ENS is the full 4-component ensemble model; IHS is the model predicting the inverse hyperbolic sine transformation of net wealth; WD is the model predicting the net difference between estimates of gross wealth and debt; GLM is the generalized linear model (logistic transformation for binary model); EN is the elastic net model; RF is the random forest; XGB is the gradient boosted trees model; NET is the artificial neural network; SVM is the support vector machine; and KNN is the K nearest neighbors model.

**Fig. 6:**
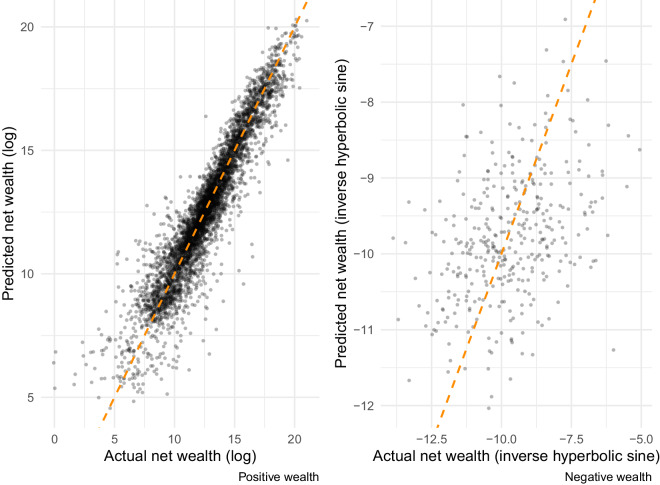
Test sample performance (SCF), positive and negative wealth stacked ensembles. Separately for households with positive and negative wealth in the SCF test-sample data (N = 5,341), this figure describes the correlation between actual and predicted values of net wealth. Root mean squared error (RMSE) for positive wealth estimate equal to 0.99 and the correlation coefficient is 0.94. RMSE for negative wealth estimates is 1.48 and the correlation coefficient is 0.38.

To visually inspect predicted versus actual values, Fig. 6 shows the performance of the full ensemble models separately for households with positive and negative wealth in the hold-out SCF data (i.e. 10% of data). These figures show the predicted and true values of net wealth for households. A line of symmetry is included for the diagonal. There is an evident strong fit for households with positive wealth (91% of the sample; *RMSE* = 0.99, *r* = 0.94) - the stacked ensemble errors are symmetrical and highly accurate at the household-level. Due to a relative lack of information in the data on liabilities, which can be used to quantify negative wealth, our estimation does not perform as well for households with negative wealth (7.5% of sample; *RMSE* = 1.48, *r* = 0.38).

Declines in predictive performance are evident in cases where income measures are coarser or when key items are missing (for example, from 1960 to 1970, rather than being separately identified, investment income is incorporated into ‘other’ income). The relative decline is, however, modest. Table 5 details year-specific performance results for our preferred (‘winning’) ensemble approach.

**Table 5:** Performance of ensemble combination by year.

Year	Brier	Kappa	AUC	Accuracy	TPR	TNR	RMSE ( + )	RMSE (−)
2010–2020	0.058	0.426	0.901–0.921	0.887	0.910	0.637	0.999	1.480
1990–2000	0.058	0.408	0.891–0.913	0.894	0.925	0.554	1.002	1.497
1980	0.063	0.379	0.878–0.901	0.861	0.881	0.653	1.058	1.432
1970	0.063	0.389	0.877–0.9	0.860	0.876	0.685	1.151	1.445
1960	0.063	0.384	0.873–0.897	0.859	0.876	0.679	1.206	1.438

Note: Using performance metrics described in the text, this table compares performance of the ensemble combinations across different years, corresponding to different variable sets available in the Census/ACS.

### Performance evaluation, out of sample dataset (PSID)

**Fig. 7:**
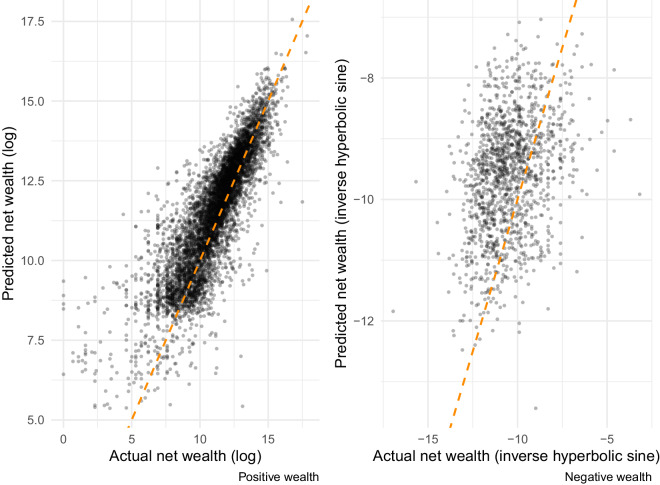
Out of sample performance (PSID), positive and negative wealth stacked ensembles. Separately for households with positive and negative wealth in 2019 Panel Study of Income Dynamics (PSID) data, this figure describes the correlation between actual net wealth and predicted values using our ensemble model. Root mean squared error (RMSE) for positive wealth estimate equal to 1.26. RMSE for negative wealth estimates is 1.75.

To further validate the performance of the ensemble models, we next evaluate model fit through an independent source of wealth data – the 2019 wave of the Panel Study of Income Dynamics (PSID). The PSID provides an identical set of variables to those used to build the models that are trained from the SCF. There is one important difference, however – the PSID definition of net wealth excludes the value of pensions while SCF includes it^62^. Figure 7 plots PSID households’ observed wealth levels against the levels of wealth predicted by our models. These plots show performance patterns that are consistent with what we observed in the test sample: strong performance in predicting positive wealth and somewhat less so for negative wealth. By comparison to the SCF test sample, the PSID-based estimates exhibit higher absolute error levels for positive and negative wealth (*RMSE* = 1.26 and *RMSE* = 1.75 respectively). This discrepancy is to be anticipated given that the PSID excludes pensions in its definition of net wealth.

Since PSID includes State identifiers, we can also assess variation in prediction errors across locations. For each ensemble, we run a simple linear regression model in which the prediction errors from our models are regressed on the state identifiers. For binomial ensemble models, the dependent variable is a binary indicator for whether prediction is accurate or inaccurate; for ensembles predicting levels of positive or negative wealth, the dependent variable is the residual difference between the predicted and the observed values. In these analyses, we find that states explain relatively little of the overall variation in erroneous predictions (adjusted-*R*-squared of less than 1%). Since neither the PSID nor any other known publicly-available data on wealth offers geographic identifiers below the level of an individual state, we are unable to directly assess our estimates of inequality at finer spatial scales. One of the main contributions of the GEOWEALTH-US database will be in enabling research into wealth dynamics at these finer spatial scales.

### Validation against aggregate published measures of wealth inequality

As a further validation exercise, we compare aggregates of our imputed Census wealth data against widely-recognized published data at national and state levels.

**Fig. 8:**
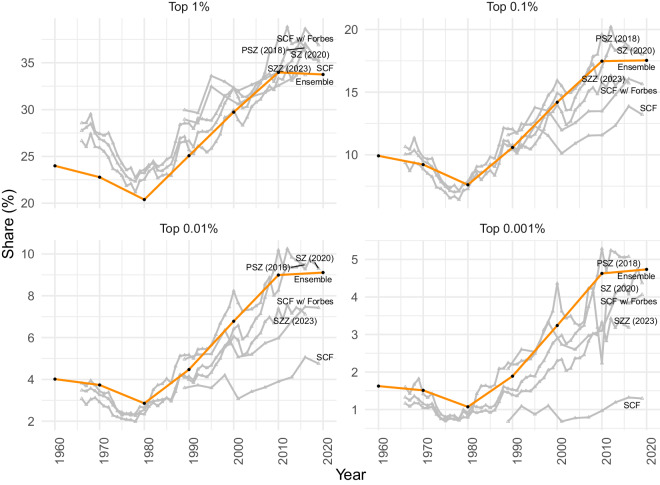
Comparing national top wealth share estimates across different measurement approaches. For six different measures, each panel tracks the share of total national wealth held by a specific top percentile of the wealth distribution. Across the panels, the different series are: ‘Ensemble’, in orange, which is the estimate generated using our ensemble model; ‘SCF’, estimated from the raw Survey of Consumer Finances;‘SCF w/Forbes’ which adds the Forbes 400 to the raw SCF; ‘PSZ (2018)’ which makes use of Piketty, Saez and Zucman’s estimates using the distributional national accounts framework^24^; ‘SZ (2020)’ which is an updated distributional macroeconomic accounts series generated by Saez and Zucman^40^; and ‘SZZ (2023)’ which uses income-capitalized estimates based on tax data from Smith, Zidar and Zwick^44^.

Regarding national estimates, Fig. 8 compares our ‘Ensemble’ approach, visualized in orange, against other measures of the share of national wealth held by a specific top percentile of wealth holders. Across each of the four panels, the other series include: ‘SCF’, estimated directly from the Survey of Consumer Finances; ‘SCF w/Forbes’, derived from a combination of the SCF and the Forbes 400 list; ‘PSZ (2018)’, referring to the estimates of Piketty, Saez and Zucman’s from the distributional national accounts framework^24^; ‘SZ (2020)’, an updated distributional macroeconomic accounts series^40^; and, finally, ‘SZZ (2023)’, which uses income-capitalized estimates based on administrative tax data from Smith, Zidar and Zwick^44^.

Figure 8 indicates that the our estimates of top wealth shares, from the ensemble approach, compare favorably to the main alternatives. There is a strong relationship between our top wealth share estimates and those derived from the national distributional accounts method. For the share of wealth held by the top one percent, this consistency extends to the SCF, as well as the combined SCF and Forbes 400. When we consider the very wealthiest households, our model produces estimates that appear increasingly distinct from the SCF, and to some extent both the SCF augmented by the Forbes ‘rich list’ and Smith, Zidar and Zwick^44^. Such differences may reflect the advances made by our approach in estimating how wealth is distributed among the very wealthiest households (see Steps 2 and 3 above). They may also result in part from differences in the units of observation under study: while SCF-based estimates, including ours, are focused on households, the national distributional accounts methods are based on (synthetic) individuals.

**Fig. 9:**
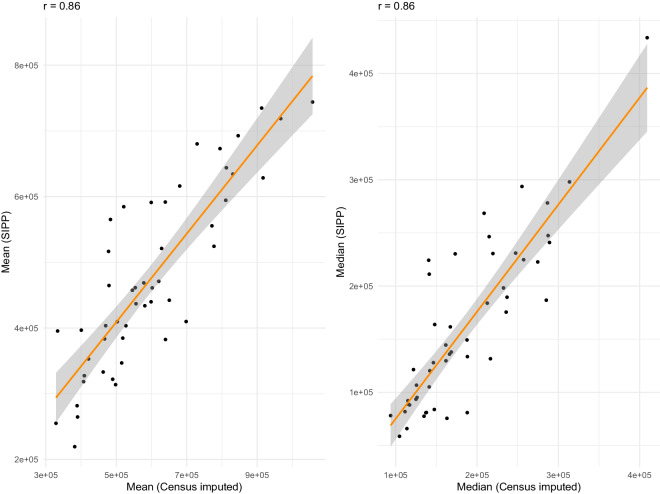
US state-level comparisons, mean and median wealth from imputed estimates and SIPP (2020). Panels compare state-level mean and median wealth, using estimates generated from our ensemble model and those obtained from the 2020 Survey of Income and Program Participation (SIPP).

Over recent years, the U.S. Census Bureau’s Survey of Income and Program Participation (SIPP) – a nationally representative survey tracking household economic outcomes and government program participation – has provided estimates of mean and median wealth at the state-level. We can thus compare our state-level estimates to those provided by SIPP. The scatterplots in Fig. 9 describe the relationship between SIPP state-level measures of mean and median wealth and our Census imputations in 2020. These measures are strongly correlated at both the mean (*r* = 0.86) and the median (*r* = 0.86). This high level of correspondence provides further validation for our imputed estimates, this time at a subnational scale.

### Descriptive analysis

Having described the process to build the GEOWEALTH-US database^29^ and our external validation procedures, we conclude this article by presenting an initial overview of some of the key patterns in the dataset that can inform future research. We do so by focusing on two separate indicators of wealth inequality, one that captures wealth concentration within commuting zones and one that measures wealth differences between commuting zones.

**Fig. 10:**
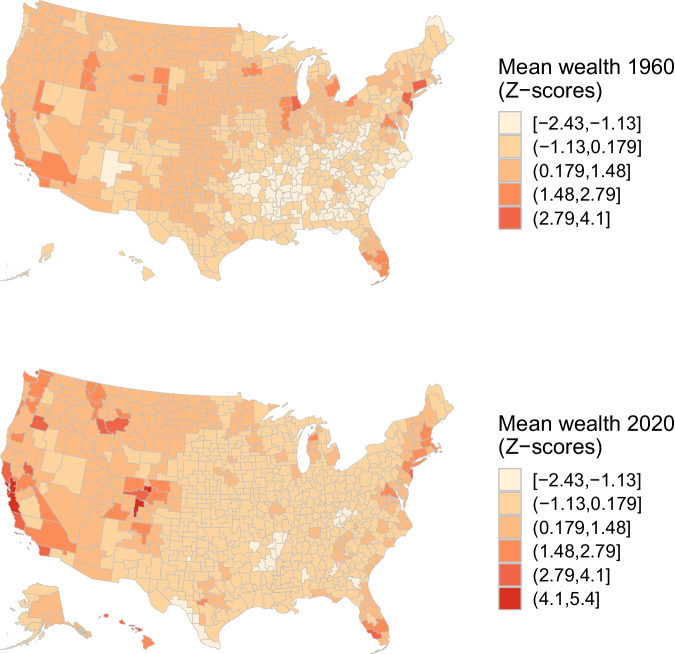
Average wealth levels across commuting zones, 1960 & 2020. For 1990-vintage commuting zones (CZs) as in Tolbert and Sizer^73^, this figure maps mean wealth levels in 1960 and 2020. Local average wealth levels are converted to z-scores such that the distribution has a mean of zero and a standard deviation equal to one.

To examine how wealth levels have changed across commuting zones over the past 60 years, Fig. 10 maps average wealth in 1960 and 2020. Given the large increases in average wealth over the study period, we standardize average wealth into period-specific *z*-scores, which have a mean of zero and a standard deviation of one. This metric provides a relative indication of how wealthier and poorer commuting zones deviate from the average for each period.

In 1960, commuting zones in traditional urban and industrial regions of the country exhibit the highest average levels of wealth. High wealth level are evident across the Northeast, the greater Chicago region, and in the Sunbelt in Southern California and Florida. These patterns closely track well known patterns of regional development in the early- to mid-twentieth century^63^.

While patterns of average wealth in 2020 bear some resemblance to those in 1960, several important differences are evident. Specifically, the advantages of many once wealthy manufacturing regions have regressed toward the mean. This is particularly notable for the metropolitan areas around the Great Lakes such as Buffalo, Cleveland, Chicago, and Milwaukee. In their place, Pacific cities such as Seattle, Los Angeles, San Francisco, interior regions like Denver, and the major Texan metropolises have decidedly improved their relative wealth positions. Although the South as a whole continues to lag the rest of the country in terms of average wealth, Savannah (GA), Raleigh (NC), and Nashville (TN) are examples of Southern commuting zones that have seen substantial increases in their average wealth levels.

**Fig. 11:**
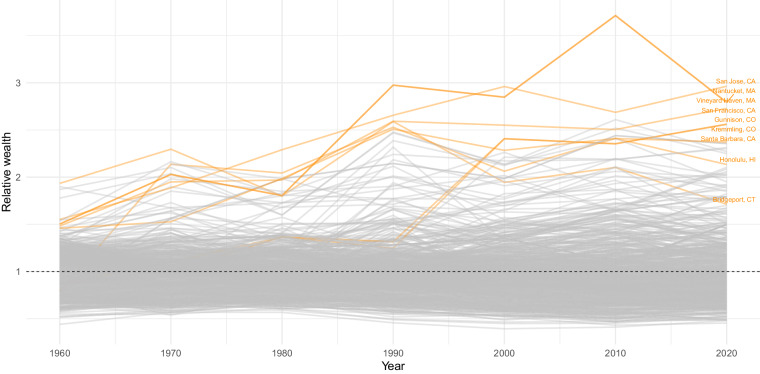
Relative wealth over time, U.S. Commuting Zones. Each line in this figure represents a particular U.S. Commuting Zone (CZ), defined according to 1990 boundaries as per Tolbert and Sizer^73^. In each observed year, the Y-axis measures the ratio of average wealth for a CZ to the all-locations average wealth.

As noted above, the changing geography of wealth over this period is also characterized by an intensification of inequality between regions. This is evident in Fig. 11, where we plot the trajectories of relative wealth for commuting zones across each decade. Most clearly, this figure exhibits a pattern of fanning out, implying rising levels of inter-regional wealth inequality since 1960. This means that the average wealth gaps between the wealthiest commuting zones and the average commuting zone are significantly larger in 2020 than they were in 1960. For example, Bridgeport and Chicago, which were among the top five wealthiest commuting zones in 1960, had average wealth levels that were roughly 4 standard deviations above the average. In 2020, however, the average wealth levels of San Jose and San Francisco - two of the wealthiest commuting zones today - are 5 standard deviations above the mean. Preliminary investigation of the GEOWEALTH-US database therefore reveals that the wealthiest regions have been pulling away from the rest of the country since 1960.

**Fig. 12:**
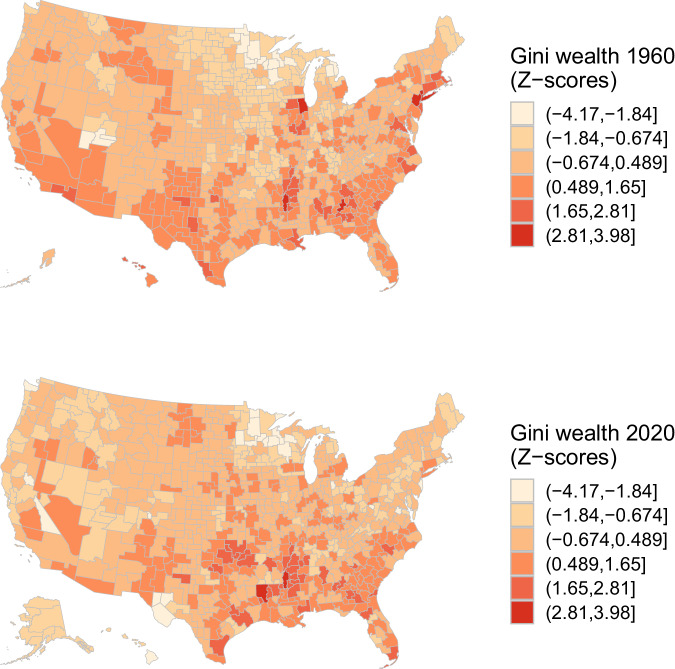
Gini coefficients for wealth distribution within commuting zones, 1960 & 2020. For 1990-vintage commuting zones (CZs) as in Tolbert and Sizer^73^, this figure maps Gini coefficients *Z*-scores tracking within-CZ wealth inequality in 1960 and 2020.

Finally, we turn our attention to the changing dynamics of wealth inequality within regions over time. Figure 12 maps the Gini coefficiens (*z*-scores) for wealth inequality within commuting zones in 1960 and 2020, revealing patterns of change and stability. In 1960, intra-regional wealth was high throughout the South, low in the Midwest and Northern Plains regions, and more mixed along the coasts. The main change to this pattern up to 2020 has, however, been the dramatic rise in inequality in the Midwest and Plains regions. While the South persists as a region that broadly exhibits high inequality, central and formerly manufacturing-dependent Midwestern regions have seen a substantial worsening of inequality over this period. This convergence in terms of inequality levels between the South and the Midwest is consistent with findings from other studies of earnings inequality and intergenerational mobility^7,8^.

The GEOWEALTH-US database^29^ is thus a data compendium that can advance the frontier of social science of topics relating to economic inequality and prosperity. Over recent years, our ability to study long-term patterns of spatial inequality has been greatly enhanced through an ongoing revolution in historical data. Recent studies have generated databases that track leading indicators of inequality such as urbanization, patenting, incomes, and intergenerational mobility within consistent spatial units over long periods of time^7,64–68^. In the same vein, complementing new work on the geography of wealth during 19th and early 20th century^69^, the GEOWEALTH-US database provides a major step toward understanding the long-term spatial dynamics of wealth inequality.

## Usage Notes

We view this dataset as a necessary first step towards the study of spatial wealth disparities. While one would ideally want directly observed, geocoded data on household wealth in the United States and how it has changed, such data are unlikely to become widely available in the near future. The only known precursor to the data described in this article is Chenevert *et al*.^70^ which describes preliminary attempts to build State-specific measures of wealth inequality using regression-based imputation. GEOWEALTH-US data^29^ could serve as a foundation to explore a wide range of questions on the causes and consequences of changing spatial patterns of wealth and wealth inequality, which heretofore have been difficult to explore. Just as there is a growing literature on community and spatial effects of differences in income and poverty, these data provide a basis to answer related questions around wealth.

Potential users of these data should note that, although the data underlying this study enumerate characteristics of human subjects and their households, they have been fully anonymized by the agencies responsible for the data. The study nonetheless obtained approval from the Social Sciences, Humanities & Education Research Ethics Board at the University of Toronto.

## Data Availability

All replication code is available at https://github.com/jhsuss/wealth-inequality.

## References

[1] Kuhn M, Schularick M, Steins UI (2020). Income and wealth inequality in America, 1949–2016. Journal of Political Economy.

[2] Saez E, Zucman G (2016). Wealth inequality in the United States since 1913: Evidence from capitalized income tax data. The Quarterly Journal of Economics.

[3] Piketty, T. & Zucman, G. Wealth and inheritance in the long run. In Atkinson, A. & Bourguignon, F. (eds.) *Handbook of income distribution*, vol. 2, 1303–1368 (Elsevier, 2015).

[4] Goldin, C. & Katz, L. F. *The race between education and technology* (Harvard University Press, 2009).

[5] Song X (2020). Long-term decline in intergenerational mobility in the united states since the 1850s. Proceedings of the National Academy of Sciences.

[6] Sampson R (2019). Neighbourhood effects and beyond: Explaining the paradoxes of inequality in the changing American metropolis. Urban Studies.

[7] Connor DS, Storper M (2020). The changing geography of social mobility in the United States. Proceedings of the National Academy of Sciences.

[8] Kemeny, T. & Storper, M. The changing shape of spatial income disparities in the United States. *Economic Geography* 1–30 (2023).

[9] Connor, D. S., Berg, A. K., Kemeny, T. & Kedron, P. J. Who gets left behind by left behind places? *Cambridge Journal of Regions, Economy and Society*, rsad031 (2023). 10.1093/cjres/rsad031 PMC10928807 38482342

[10] Connor, D. S., Hunter, L., Jang, J. & Uhl, J. H. Family, community, and the rural social mobility advantage. *Research in Social Stratification and Mobility*, **87**, 100844 (2023). 10.1016/j.rssm.2023.100844 PMC10829533 38304057

[11] Connor, D. S., Kemeny, T. & Storper, M. Frontier workers and the seedbeds of inequality and prosperity. *Journal of Economic Geography*, lbad018 (2023).

[12] Neckerman KM, Torche F (2007). Inequality: Causes and consequences. Annu. Rev. Sociol..

[13] Yellen, J. L. Perspectives on inequality and opportunity from the Survey of Consumer Finances Speech to the Conference on Economic Opportunity and Inequality, Federal Reserve Bank of Boston (2014).

[14] Côté S, House J, Willer R (2015). High economic inequality leads higher-income individuals to be less generous. Proceedings of the National Academy of Sciences.

[15] Baumgärtner S, Drupp MA, Meya JN, Munz JM, Quaas MF (2017). Income inequality and willingness to pay for environmental public goods. Journal of Environmental Economics and Management.

[16] Hansen MN (2014). Self-made wealth or family wealth? Changes in intergenerational wealth mobility. Social Forces.

[17] Acolin A, Wachter S (2017). Opportunity and housing access. Cityscape.

[18] Chetty, R., Friedman, J. N., Saez, E., Turner, N. & Yagan, D. Mobility report cards: The role of colleges in intergenerational mobility. National Bureau of Economic Research Working Paper 23618 (2017).

[19] Cramer, K. J. *The politics of resentment: Rural consciousness in Wisconsin and the rise of Scott Walker* (University of Chicago Press, 2016).

[20] Rodrguez-Pose A (2018). The revenge of the places that don’t matter (and what to do about it). Cambridge Journal of Regions, Economy and Society.

[21] Broz JL, Frieden J, Weymouth S (2021). Populism in place: the economic geography of the globalization backlash. International Organization.

[22] Moretti E (2010). Local multipliers. American Economic Review.

[23] Couture, V., Gaubert, C., Handbury, J. & Hurst, E. Income growth and the distributional effects of urban spatial sorting. National Bureau of Economic Research Working Paper 26142 (2019).

[24] Piketty T, Saez E, Zucman G (2018). Distributional national accounts: methods and estimates for the United States. The Quarterly Journal of Economics.

[25] Pfeffer FT, Waitkus N (2021). The wealth inequality of nations. American Sociological Review.

[26] Killewald A, Pfeffer FT, Schachner JN (2017). Wealth inequality and accumulation. Annual Review of Sociology.

[27] Cowell, F., Nolan, B., Olivera, J. & Van Kerm, P. Wealth, top incomes and inequality. In Hamilton, K. & Cameron, H. (eds.) *National Wealth: What is missing, why it matters*, 175–206 (New York: Oxford University Press, 2017).

[28] Auten, G. & Splinter, D. Income inequality in the United States: Using tax data to measure long-term trends. *Journal of Political Ecomomy*. (Accepted) (2023).

[29] Suss J, Kemeny T, Connor D (2024). ICPSR.

[30] CID. The Stone Center for Inequality Dynamics. https://www.inequalitydynamics.umich.edu [Accessed: 11/30/2023] (2023).

[31] Gyourko J, Mayer C, Sinai T (2013). Superstar cities. American Economic Journal: Economic Policy.

[32] Ganong P, Shoag D (2017). Why has regional income convergence in the US declined?. Journal of Urban Economics.

[33] CBO. Trends in the distribution of family wealth, 1989 to 2019. Tech. Rep., Congressional Budget Office (2022).

[34] O’Brien, D. T. *Urban Informatics: Using Big Data to Understand and Serve Communities* (CRC Press, 2022).

[35] Chetty R (2021). Improving equality of opportunity: New insights from big data. Contemporary Economic Policy.

[36] Manduca RA (2019). The contribution of national income inequality to regional economic divergence. Social Forces.

[37] Ruggles, S., Flood, S., Goeken, R., Schouweiler, M. & Sobek, M. IPUMS USA: Version 12.0 [dataset]. Minneapolis, MN, 10.18128/D010.V12.0 (2022).

[38] Combes P-P, Duranton G, Gobillon L (2008). Spatial wage disparities: Sorting matters!. Journal of Urban Economics.

[39] Vermeulen P (2016). Estimating the top tail of the wealth distribution. American Economic Review.

[40] Saez E, Zucman G (2020). The rise of income and wealth inequality in america: Evidence from distributional macroeconomic accounts. Journal of Economic Perspectives.

[41] Bricker, J., Henriques Volz, A. & Hansen, P. How much has wealth concentration grown in the United States? a re-examination of data from 2001–2013. FEDS Working Paper No. 2018-024 (2018).

[42] Kennickell AB (2017). Getting to the top: Reaching wealthy respondents in the SCF. Statistical Journal of the IAOS.

[43] Vermeulen P (2018). How fat is the top tail of the wealth distribution?. Review of Income and Wealth.

[44] Smith M, Zidar O, Zwick E (2023). Top wealth in america: New estimates under heterogeneous returns. The Quarterly Journal of Economics.

[45] Breiman L (1996). Stacked regressions. Machine Learning.

[46] Zhou, Z.-H. *Ensemble methods: foundations and algorithms* (CRC press, 2012).

[47] Breiman L (1996). Bagging predictors. Machine learning.

[48] Hastie, T., Tibshirani, R., Friedman, J. H. & Friedman, J. H. *The elements of statistical learning: data mining, inference, and prediction*, vol. 2 (Springer, 2009).

[49] Lundberg, S. M. & Lee, S.-I. A unified approach to interpreting model predictions. *Advances in Neural Information Processing systems* **30** (2017).

[50] Burkhauser RV, Feng S, Jenkins SP, Larrimore J (2011). Estimating trends in US income inequality using the Current Population Survey: The importance of controlling for censoring. The Journal of Economic Inequality.

[51] Fichtenbaum R, Shahidi H (1988). Truncation bias and the measurement of income inequality. Journal of Business & Economic Statistics.

[52] Chen, T. & Guestrin, C. Xgboost: A scalable tree boosting system. In *Proceedings of the 22nd ACM SIGKDD International Conference on Knowledge Discovery and Data Mining*, 785–794 (2016).

[53] Burges CJ (2010). From ranknet to lambdarank to lambdamart: An overview. Learning.

[54] Jenkins SP (2017). Pareto models, top incomes and recent trends in uk income inequality. Economica.

[55] Cowell FA, Flachaire E (2007). Income distribution and inequality measurement: The problem of extreme values. Journal of Econometrics.

[56] Piketty T (2003). Income inequality in france, 1901–1998. Journal of Political Economy.

[57] Atkinson AB, Piketty T, Saez E (2011). Top incomes in the long run of history. Journal of Economic Literature.

[58] Dalitz, C. Estimating wealth distribution: Top tail and inequality. *arXiv preprint arXiv:1807.03592* (2018).

[59] Bricker J, Henriques A, Krimmel J, Sabelhaus J (2016). Measuring income and wealth at the top using administrative and survey data. Brookings Papers on Economic Activity.

[60] Dorn, D. *Essays on inequality, spatial interaction, and the demand for skills*. Ph.D. thesis, University of St. Gallen (2009).

[61] Efron, B. *Bootstrap methods: Another look at the jackknife* (Springer, 1992).

[62] Cooper, D., Dynan, K. E. & Rhodenhiser, H. Measuring household wealth in the Panel Study of Income Dynamics: The role of retirement assets. Federal Reserve Bank of Boston Working Paper (2019).

[63] Lindert, P. H. & Williamson, J. G. *Unequal Gains: American Growth and Inequality since 1700* (Princeton University Press, 2017).

[64] Leyk S (2020). Two centuries of settlement and urban development in the United States. Science Advances.

[65] Uhl, J. *et al*. Place-level urban–rural indices for the United States from 1930 to 2018. *Landscape and Urban Planning* **236** (2023). 10.1016/j.landurbplan.2023.104762 PMC10310068 37396149

[66] Uhl J, Connor D, Leyk S, Braswell A (2021). A century of decoupling size and structure of urban spaces in the United States. Communications Earth & Environment.

[67] Petralia, S., Balland, P. A. & Rigby, D. L. Unveiling the geography of historical patents in the United States from 1836 to 1975. *Scientific Data* **3** (2016). 10.1038/sdata.2016.74 PMC5004586 27576103

[68] Bauluz, L. *et al*. Spatial wage inequality in north america and western europe: changes between and within local labour markets 1975–2019. Kiel Working Paper, No. 2253 (2023).

[69] Dray, S., Landais, C. & Stantcheva, S. Wealth and property taxation in the United States. National Bureau of Economic Research Working Paper 31080 (2023).

[70] Chenevert, R., Gottschalck, A., Klee, M. & Zhang, X. Where the wealth is: the geographic distribution of wealth in the United States. Social, Economic and Housing Statistics Division, US Census Bureau (2017).

[71] Kennickell, A. B. Multiple imputation in the survey of consumer finances. In *Proceedings of the Section on Survey Research Methods* (1998).

[72] Kennickell AB (2017). Wealth measurement in the survey of consumer finances: Methodology and directions for future research. Statistical Journal of the IAOS.

[73] Tolbert, C. M. & Sizer, M. US commuting zones and labor market areas: A 1990 update. United States Department of Agriculture, Staff report (1996).

